# Safety Evaluation of an Expedited Omalizumab Home Self-Administration Pathway

**DOI:** 10.7759/cureus.19434

**Published:** 2021-11-10

**Authors:** Zobia Hussain, Lisa Devlin

**Affiliations:** 1 Emergency Medicine, Royal Victoria Hospital, Belfast, GBR; 2 Allergy and Immunology, Royal Victoria Hospital, Belfast, GBR

**Keywords:** covid-19, anaphylaxis, patient safety, self-administration, service provision, chronic spontaneous urticaria, omalizumab, immunology

## Abstract

Introduction

The Northern Ireland Regional Immunology Service (NIRIS) has developed an expedited omalizumab home self-administration pathway to reduce face-to-face clinic attendance during the coronavirus disease 2019 (COVID-19) pandemic. This audit evaluates the safety of this pathway with a particular focus on anaphylaxis.

Objectives

This study aimed to retrospectively audit the records of 39 patients undertaking expedited home self-administration at NIRIS for complications, particularly emergency department attendance for anaphylaxis. The target was for 100% of patients to complete a six-month course without experiencing anaphylaxis related to omalizumab administration.

Materials and methods

A total of 39 records of patients who underwent expedited omalizumab self-administration were audited by a single reviewer. They were prospectively collected between March 2020 and August 2021. Clinical data were collected from the Northern Ireland Electronic Care Record (NIECR).

Results

Hundred percent of patients were in the process of completing or had completed a six-dose course without anaphylaxis. During the course of omalizumab, 7.6% of patients attended the emergency department. Zero percent of patients have experienced anaphylaxis triggered by omalizumab. The target of 100% patients completing the expedited pathway without omalizumab-related anaphylaxis was met.

Conclusion

Home self-administration of omalizumab is preferred by patients and clinicians for reducing expense, travel, and unnecessary clinical contact during the COVID-19 pandemic. An expedited omalizumab home self-administration training pathway appears to be safe in a population of Northern Irish patients with chronic spontaneous urticaria (CSU). More research is needed to determine whether the expedited pathway should become the standard of care post-pandemic

## Introduction

Omalizumab (Xolair©) is an anti-IgE monoclonal antibody used as adjunctive therapy in the management of chronic spontaneous urticaria (CSU) and moderate-to-severe allergic asthma [1]. As a CSU treatment, it is administered as a subcutaneous injection once every four weeks for a course of six treatments. These treatments were previously only licensed for administration by a healthcare professional under medical supervision due to a perceived risk of anaphylaxis, although the documented risk in the literature was low (0.1%) [2]. 

The coronavirus disease 2019 (COVID-19) pandemic significantly disrupted healthcare, and services were required to reduce face-to-face attendances in order to decrease transmission of the virus. The British Society of Allergy and Clinical Immunology (BSACI) published recommendations relating to home therapy included expediting home self-administration pathways and extra-license usage of home self-administration in patients with a history of anaphylaxis which had a clear trigger unrelated to omalizumab [3]. The Northern Ireland Regional Immunology Service has developed an omalizumab home self-administration pathway in which patients receive four doses under medical supervision with training on how to self-administer, followed by home self-administration for the remainder of the course. Weighing up the low risk of anaphylaxis against the benefit of reducing footfall during the pandemic, it was determined that new patients may receive one treatment and training under medical supervision followed by home self-administration instead of the standard four doses. This safety audit investigates the safety of the expedited omalizumab home self-administration pathway.

## Materials and methods

This is a retrospective audit assessing the total number of patients who underwent expedited omalizumab self-administration between March 2020 and August 2021 (N=39). Patients were identified by the clinical coordinator from a departmental spreadsheet. Clinical data were collected from the Northern Ireland Electronic Care Record (NIECR). Data were put into and analysed using Microsoft Excel by a medical student. The objective of this study was to identify the incidence of anaphylaxis in patients on the expedited omalizumab home self-administration pathway. The standardised procedure followed in omalizumab home self-administration training is mentioned in Appendices. The audit standards are mentioned in Table 1 below.

**Table 1:** Omalizumab home self-administration safety audit standards BSACI: British Society of Allergy and Clinical Immunology; COVID-19: coronavirus disease 2019

Audit criteria	Target (%)	Exceptions	Source of evidence
Patients receiving expedited omalizumab home self-administration therapy commence a six-dose course without experiencing omalizumab-related anaphylaxis (criterion 1)	100	Nil	BSACI: modifications for adult allergy services during the COVID-19 pandemic

## Results

The results related to criterion 1 are mentioned in Table 2 below. Hundred percent (39/39) of patients were in the process of completing or had completed a six-dose course without complications. Zero out of 39 (0%) attended experienced anaphylaxis triggered by omalizumab. The target of 100% patients completing the expedited pathway without omalizumab-related anaphylaxis was met.

**Table 2:** Percentage of patients meeting audit target Criterion 1 - patients receiving expedited omalizumab home self-administration therapy commence a six-dose course without experiencing anaphylaxis.

Target	Total patients	Patients meeting criterion 1	Percentage patients meeting target	Was the target met?
100%	39	39	100%	Yes

Three out of 39 (7.6%) attended the emergency department during the course of their treatment with omalizumab. One of these patients attended because of visual disturbances unrelated to omalizumab. The second was receiving omalizumab for eight weeks for a year prior to attending the emergency department with a widespread rash, no associated airway compromise or hypotension; it was determined not to be anaphylaxis and the patient continued their course of omalizumab without issue. The third patient had already received two doses of omalizumab without issue, once under clinical supervision and once self-administered at home. Approximately two weeks after self-administering the third dose, the patient experienced a flare of their urticaria with lip swelling and throat tightening. This patient was treated with antihistamines, oral corticosteroids, and discharged; they received a subsequent dose of omalizumab without issue. The flare which occurred after the third dose suggests treatment failure. We can conclude however that this incident was not related to anaphylaxis. Patients with omalizumab-related anaphylaxis would present with hypotension in addition to airway obstruction, it is also extremely unlikely to occur after receiving a third dose when the previous two doses were well-tolerated. Hence, due to the timing and nature of the symptoms, it was determined to be a flare of CSU rather than anaphylaxis.

## Discussion

The requirement to administer omalizumab only under medical supervision causes significant disruption to patients’ daily activities, particularly for patients commuting long distances to a regional centre for each treatment [4]. In late 2018, omalizumab was approved for home self-administration in patients without a history of anaphylaxis, which bypasses some of these issues. Timmermann and Mailänder conducted a survey of patients being treated with omalizumab in 2020 and found that the majority of patients were in favour of home self-administration, with time efficiency being the primary advantage identified [4]. In simulations carried out by Shaker et al., there was a significant difference in cost-effectiveness of in-clinic omalizumab administration compared to home self-administration, with the latter being more cost-effective [5]. These findings are well supported in the literature [2,6-8]. Ninety-three percent of patients favour home self-administration which further supports the benefits of the expedited pathway [9]. 

While previous debate hinged primarily on the perceived risk of anaphylaxis, the COVID-19 pandemic pushed the issue of reducing the clinic burden to the forefront. Telehealth became increasingly important due to social distancing regulations. A 2020 systematic review found that virtual clinics improved the provision of healthcare and reduced the transmission of the virus during the outbreak [10]. Studies found a mixed patient response to pandemic telehealth measures [11-13] but found robust evidence concerning safety in light of the pandemic [14-16]. 

In light of evidence suggesting that face-to-face clinics could be safely cut down without compromising clinical care, BSACI published recommendations on strategies to minimise clinic footfall. Recommendations included deferring new treatment courses of omalizumab until the pandemic subsided, increasing the interval between doses, or discontinuing the treatment in patients partway through a course. Previously strict guidelines were loosened to allow extra-license usage of home self-administration in patients with a history of anaphylaxis which had a clear trigger unrelated to omalizumab or in shortening the pathway [3]. The effects of the pandemic and these guidelines in the real world were investigated by Krishna et al. [17]. It was found that 86-92% of immunology services continued routine appointments including new referrals but most of these consultations were telehealth or virtual, as there was a significant reduction in face-to-face consultations amongst the 99 immunology and allergy services participating in the study. The vast majority of services changed immunoglobulin administration regimens and reduced sublingual and subcutaneous injection immunotherapy among other therapeutic procedures. Adverse outcomes relating to these changes primarily centred around the immunoglobulin replacement therapy, although some centres reported exacerbations of CSU relating to missed omalizumab doses [17]. One flaw of these reports is inconsistent coding of anaphylaxis and urticarial symptoms; it is possible that the incidents of urticaria may have been misdiagnosed as anaphylaxis, falsely raising the rate of this complication. The Northern Ireland Regional Immunology Service (NIRIS) did not report any such adverse events owing in large part to the timely expedited home self-administration pathway, which successfully allowed continuing treatment alongside reducing the risk of COVID-19 spread. Indeed, from the reports in the literature on similar practices elsewhere, it appears that the risk of anaphylaxis may be overblown and that the benefits of empowering patients to take charge of their care may continue to benefit them in post-pandemic [17]. 

Limitations

The primary limitations of this study centre around the small sample size of patients investigated (N=39). This audit should be repeated in six to 12 months to validate previous results and investigate temporal trends. It is possible that a degree of selection bias may be present in those patients who choose to undergo home self-administration training may be less likely to experience anaphylaxis; however, anaphylaxis is unrelated to lifestyle factors so this bias is unlikely. The population of Northern Irish CSU patients may have different characteristics from the larger population of CSU patients. More research is needed to conclusively determine the safety of this approach.

## Conclusions

Although omalizumab home self-administration is considered controversial due to a perceived risk of anaphylaxis, the results of this audit suggest that allowing Northern Irish patients to complete the pathway after a single supervised treatment is safe. There was a clinical need for an expedited self-administration pathway during the COVID-19 pandemic, and the expedited pathway was an appropriate measure to reduce face-to-face interaction, hence reducing transmission of the virus. It is plausible that this expedited pathway could be continued after pandemic restrictions are lifted, which could be beneficial to both patients and clinicians by increasing time efficiency. More research is needed before generalising these findings to other patient populations.

## Figures and Tables

**Figure 1:**
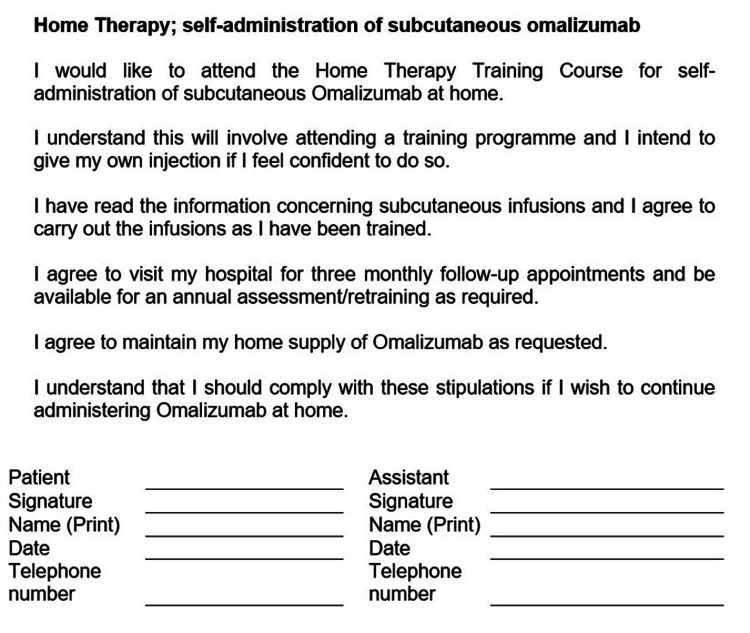
Omalizumab home administration consent form

**Figure 2:**
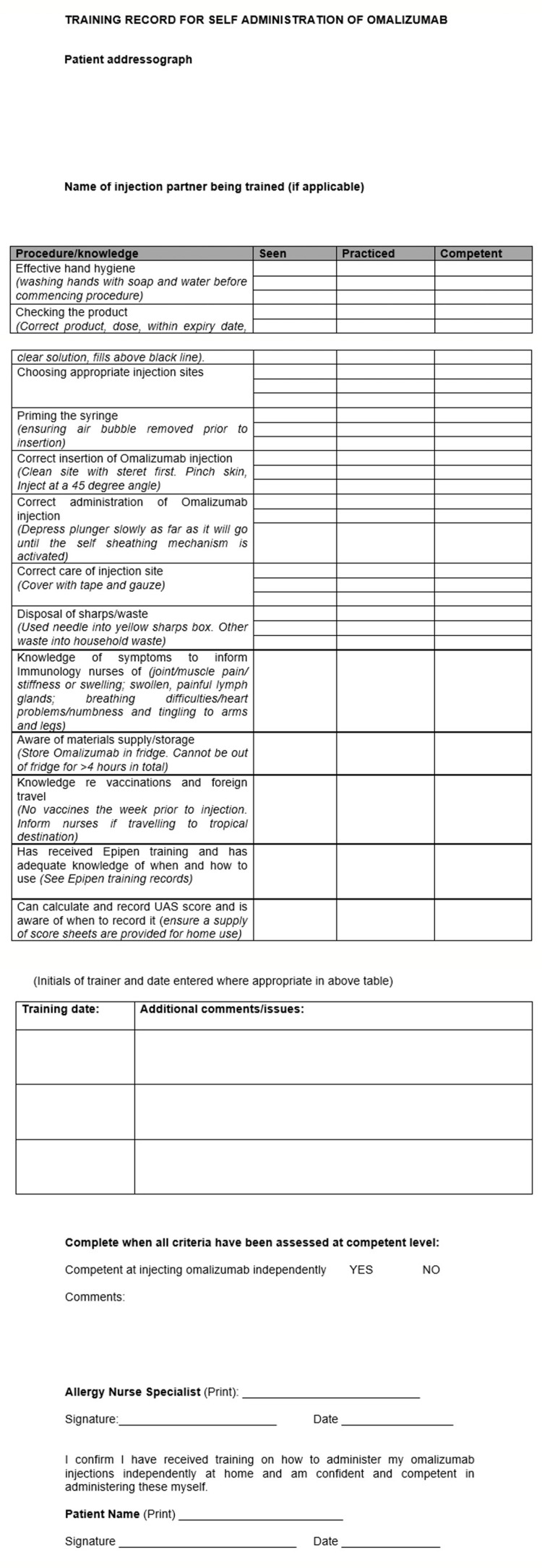
Omalizumab home self-administration training record UAS: urticaria activity score

## Appendices

Standard 4.2.1 training procedure for self-administration of omalizumab

4.2.1.a: Introduction

Selected patients who are established on omalizumab for chronic spontaneous urticaria will be given the option to be trained for self-administration within the home setting.

4.2.1.b: Scope and Purpose

This document outlines the procedure to be followed in selecting and training patients.

4.2.1.c: Related Documents

1. Standard 4.2.2 Omalizumab (Xolair) for the treatment of Chronic Spontaneous Urticaria

2. Sign 153 - British guideline on the management of asthma, September 2016

4.2.1.d: Procedure

(A) Selection of patients for self-administration of omalizumab

1. All patients must have had four doses of omalizumab administered in the hospital setting without any adverse effect, such as urticaria; angioedema; respiratory distress - wheeze, dyspnoea, tachypnoea; vomiting, diarrhoea, severe abdominal discomfort.

2. Patients must consent to train

3. All patients with asthma/COPD should be discussed with a member of the medical team before training for self-administering omalizumab.

4. Patients with poorly controlled or severe asthma will be excluded from self-administering omalizumab: (a) in adults: FEV1 < 70% of the predicted value after adequate pharmacologic treatment; (b) patients on high dose therapies or continuous/frequent use of oral corticosteroids (see SIGN 154 British guidelines on the management of asthma)

(B) Consent for home administration of omalizumab - agreement form (Figure 1)

(C) Training procedure for injection of omalizumab

1. Equipment required - clean tray or tabletop (wiped down with steret); alcohol skin wipes; plaster; sharps box; waste-paper bin; omalizumab injection

2. Method - (i) wash and dry hands; (ii) remove the syringe from the refrigerator 30 minutes before administration to allow it to reach room temperature. If not administered the syringe can be returned to the fridge ONCE only.  However, the time out of the fridge must not exceed four hours; (iii) assemble equipment in a clean area; (iv) clean area of skin with an alcohol wipe; (v) holding the syringe with the needle pointing upwards, carefully pull off the needle cap from the syringe and discard it. Do not touch the exposed needle. Then, gently tap the syringe with your finger until the air bubble rises to the top of the syringe. Slowly push the plunger up to force the air bubble out of the syringe without inadvertently expelling the solution. (vi) Gently pinch the skin of your upper thigh or abdomen and insert the needle; (vii) holding onto the finger flange, slowly depress the plunger as far as it will go. If any solution leaks from the injection site, insert the needle further; (viii) keeping the plunger fully depressed, carefully lift the needle straight out from the injection site; (ix) slowly release the plunger and allow the needle guard to automatically cover the exposed needle; (x) dispose of the syringe in the sharps container

(D) Training record for self-administration of omalizumab (Figure 2; see Table 3 for urticaria activity score {UAS}). 

**Table 3:** UAS once-daily version - daily scoring for itch and hives UAS: urticaria activity score

Itch severity score	Itch severity (once every 24 h)	Hives severity score	Number of hives per 24 h
0	None	0	None
1	Mild (present but not annoying or troublesome)	1	< 20
2	Moderate (troublesome but does not interfere with normal daily activity or sleep)	2	20–50
3	Intense (interferes with normal daily activity or sleep)	3	> 50
